# Advances in Sensing, Response and Regulation Mechanism of Salt Tolerance in Rice

**DOI:** 10.3390/ijms22052254

**Published:** 2021-02-24

**Authors:** Kimberly S. Ponce, Longbiao Guo, Yujia Leng, Lijun Meng, Guoyou Ye

**Affiliations:** 1 State Key Laboratory for Rice Biology, China National Rice Research Institute, Hangzhou 310006, China; kimsuazoponce@gmail.com; 2 Jiangsu Key Laboratory of Crop Genetics and Physiology/Key Laboratory of Plant Functional Genomics of the Ministry of Education/Jiangsu Key Laboratory of Crop Genomics and Molecular Breeding/Jiangsu Co-Innovation Center for Modern Production Technology of Grain Crops, Agricultural College of Yangzhou University, Yangzhou 225009, China; 3 CAAS-IRRI Joint Laboratory for Genomics-Assisted Germplasm Enhancement, Agricultural Genomics Institute in Shenzhen, Chinese Academy of Agricultural Sciences, Shenzhen 518120, China; menglijun@caas.cn (L.M.); yeguoyou@caas.cn (G.Y.); 4 Strategic Innovation Platform, International Rice Research Institute, DAPO BOX 7777, Metro Manila 1301, Philippines

**Keywords:** rice, salinity, sensing, signaling, transcription factors, osmoregulation, antioxidation, ion homeostasis

## Abstract

Soil salinity is a serious menace in rice production threatening global food security. Rice responses to salt stress involve a series of biological processes, including antioxidation, osmoregulation or osmoprotection, and ion homeostasis, which are regulated by different genes. Understanding these adaptive mechanisms and the key genes involved are crucial in developing highly salt-tolerant cultivars. In this review, we discuss the molecular mechanisms of salt tolerance in rice—from sensing to transcriptional regulation of key genes—based on the current knowledge. Furthermore, we highlight the functionally validated salt-responsive genes in rice.

## 1. Background

Soil salinity is one of the most significant abiotic stresses hampering plant growth and development, which ultimately translates to reduced crop yield. Soil salinization is exacerbated by excessive use of chemical fertilizers and soil amendments, improper drainage, and seawater ingress. It is estimated that over 6% of the world’s total land area is salt affected, of which over 12 million hectares are irrigated lands posing a serious threat to irrigated agriculture [1].

Rice, being one of the most important staple crops in the world, is crucial for food security in many Asian countries. However, it is the most salt-sensitive cereal crop, with varying responses at different growth stages [2]. It is relatively salt-tolerant at the germination, active tillering, and maturity stages, whereas it is highly sensitive at the early seedling and reproductive stages [1]. Salt sensitivity during the seedling stage often translates to reduced stand density in salt-affected paddies [3]. Meanwhile, sensitivity during the reproductive stage results in yield reduction, as attributed to spikelet sterility [4,5]. Hence, understanding how rice responds to salt stress is crucial in developing rice cultivars that could withstand salt stress.

Salinity imposes two major stresses in rice, (i) osmotic stress, and (ii) ionic stress. Osmotic stress is characterized by hyperosmotic soil solution disrupting cell turgor, similar to drought’s effect. In contrast, ionic stress is characterized by altered Na^+^ and K^+^ concentrations inside the cell, disrupting many biological processes [1]. Both osmotic and ionic stresses are perceived by membrane-bound cytosolic sensors that relay the stress signals to secondary messengers. In turn, the secondary messengers activate the protein phosphorylation cascades required for signal transduction pathways to develop salt-tolerant adaptive traits. In general, osmotic stress triggers the plant for stomatal closure, inhibiting shoot elongation. This ultimately results in reduced overall shoot growth, and, to a lesser extent, reduced root growth [6]. Meanwhile, ionic stress inhibits enzyme activity and therefore disrupts many biological processes, such as nitrogen metabolism [7,8]. Excess uptake of Na^+^ ions changes the NH_4_^+^ assimilation pathway, weakens the glutamate synthase pathway, and elevates the glutamate dehydrogenase pathway, impacting leaf senescence [8]. Thus, plants develop several adaptive mechanisms—namely, Na^+^ efflux from the roots to the rhizosphere, Na^+^ sequestration into the vacuole, and Na^+^ loading and unloading at the xylem—to avert the deleterious effect of Na^+^ ions in the cytosol. These mechanisms are mediated by several ion transporters coupled with H^+^-pumps. 

In the last decades, a large number of salt-responsive genes have been functionally validated in rice (Table 1). However, the overall gene regulatory network of rice responses to salt stress remains elusive. In this review, we aim to discuss the current research progress in gene regulatory networks involved in the development of salt tolerance adaptive mechanisms in rice. We also highlight the key genes involved in salt stress sensing, signaling, transcriptional regulation, and genes encoding downstream functional molecules. 

## 2. Salt Stress Sensing

**Figure 1:**
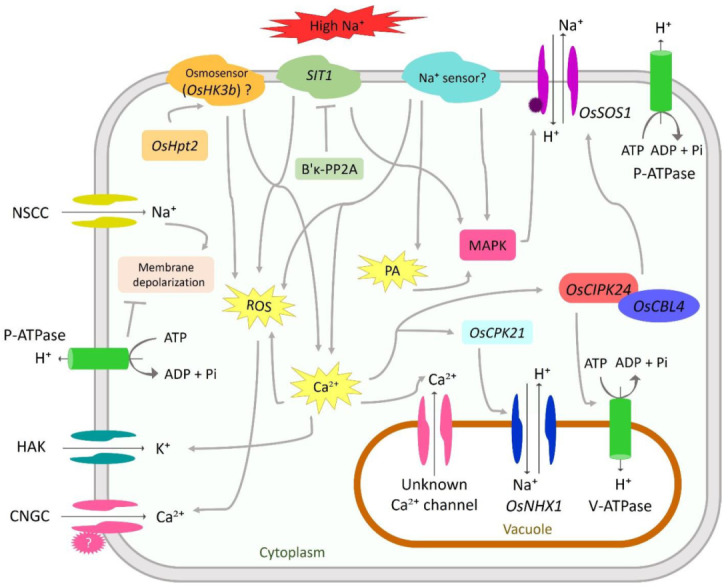
Salt sensing and signaling involved in rice responses to salt stress. Under high salinity, salt-induced osmotic stress begins, which is sensed by putative osmosensor *OsHK3b*, activated by *OsHpt2*. *SIT1* also acts as a sensor via elevated kinase activity and induces reactive oxygen species (ROS) production and mitogen-activated protein kinase (MAPK) signaling. The activity of *SIT1* is deactivated by the B’κ-PP2A subunit. Later, ionic stress occurs and is sensed by an unknown Na^+^ sensor. The Na^+^ enters the mature epidermal cell through nonselective cation channel (NSCC), causing membrane depolarization, and is polarized by P-type ATPases. Excess salt triggers a spike in the concentration of cytosolic secondary messengers, including Ca^2+^, reactive oxygen species (ROS), and phosphatidic acid (PA). ROS triggers Ca^2+^ influx through the cyclic nucleotide-gated ion channel (CNGC), activated by an unknown molecule. Ca^2+^ not only decreases K^+^ efflux but also induces further ROS accumulation; thus, a positive feedback loop exists between Ca^2+^ and ROS. The cytosolic Ca^2+^ also induces vacuolar Ca^2+^ release and activates Ca^2+^-binding proteins, such as *OsCIPK24-OsCBL4* complex. This complex, together with MAPK, activated by phosphatidic acid, upregulates the *OsSOS1* to remove cytosolic Na^+^. The vacuolar *OsNHX1* gene is activated by *OsCPK21*, whereas the V-type ATPase is activated by *OsCIPK24*, establishing a proton gradient and driving the activity of *OsNHX1*.

Stress sensing is the first event in plant response to any abiotic stresses, mounting an effective adaptive strategy. Under salt stress condition, it is presumed that osmotic and ionic stresses are perceived by membrane-bound cytosolic sensors that ultimately trigger early salt-stress signaling routes (Figure 1). However, the current knowledge of how rice sense salt stress is still limited and therefore remains an open question.

### 2.1. Osmosensing

Sensing salt-induced osmotic stress is crucial in early signaling cascades to develop salt tolerance adaptive traits, such as growth retardation, reduction in stomatal conductance, and high abscisic acid (ABA)accumulation. However, little is known about the genetics and physiology of how rice sense hyperosmotic stress. 

The transmembrane-protein-receptors, such as histidine kinases and receptor-like kinases (RLKs), function in osmotic stress perception in rice. Histidine kinases perceive osmotic fluctuations and relay the signal to response regulators via phosphotransfer, which is mediated by histidine-containing phosphotransfer protein (HpT) [101]. The first evidence of osmosensing function of histidine kinases was reported in *Arabidopsis*. The *AtHK1,* a histidine kinase encoding gene, interacts with *AtHPt1* and functions as an osmosensor during both drought and salt stress [102,103]. The ortholog of *AtHK1* in rice, *OsHK3b*, interacts with *OsHpt2* and acts as a putative osmosensor [101,104]. However, functional evidence on its osmosensing role in rice is not yet reported.

The RLKs function in drought and salt stress sensing by transmitting signals to downstream signaling pathways [105]. The rice *Salt Intolerance 1* (*SIT1*), a lectin RLK expressed mainly in root epidermal cells, acts as an upstream mediator of salt stress via elevated kinase activity [9]. Recently, Zhao et al. [106] reported that *SIT1* phosphorylates B’κ at Ser_402_, which in turn promotes the assembly of B’κ-protein phosphatase 2A (B’κ-PP2A) holoenzyme. The B’κ-PP2A subunit positively regulates salt tolerance by deactivating the activity of *SIT1* via dephosphorylation at the Thr_515/516_. *SIT1* kinase activity in turn activates the mitogen-activated protein kinase (MAPK) 3 and MAPK 6 [9]. Thus, it could be pointed out that RLKs are important in MAPK cascade activation during osmotic stress. However, the relationship between the RLKs and MAPKs needs to be further elucidated. 

Ca^2+^ permeable stress-gated cation channels (OSCA) also act as hyperosmotic stress sensors. The first evidence of the role of OSCA in osmosensing was reported in *Arabidopsis* with the characterization of *OSCA1*. The *OSCA1* gene forms a hyperosmolality-gated Ca^2+^ permeable channel during osmotic stress, thereby increasing the cytosolic free Ca^2+^ concentration [107]. The rice genome consists of 11 OSCA genes, of which seven *(OsOSCA1.1*, *OsOSCA1.2*, *OsOSCA2.1, OsOSCA2.4*, *OsOSCA2.5*, *OsOSCA3.1*, and *OsOSCA4.1*) were upregulated during salt-induced osmotic stress and may function as an osmosensor [108]. However, the Ca^2+^ conducting function of the rice OSCA genes in response to hyperosmotic stress remains an open question. 

### 2.2. Na^+^ Sensing 

The molecular mechanism of Na^+^ transport in plants is well understood; however, Na^+^ sensing remains elusive. It has been reported that the ion transporters at the plasma membrane are potential Na^+^ sensors. For instance, the plasma membrane Na^+^/H^+^ antiporter *SOS1* (*Salt Overly Sensitive 1*) is thought to be involved in Na^+^ sensing [109]. It was later proposed that only the long hydrophilic cytoplasmic tail of *SOS1* could potentially sense Na^+^ ions [110]. However, no research experiments have been undertaken to support this hypothesis, and therefore it needs to be clarified. Moreover, it is unlikely that *SOS1* functions as initial Na^+^ sensor since the *SOS3/SOS2* complex regulates its activity. Na^+^ ions could also be sensed either extracellularly and intracellularly by membrane receptors and unknown cytosolic sensors, respectively [110]. In rice, it was suggested that the intracellular Na^+^ ions are sensed by an unknown cytosolic sensor based on the observed elevated levels of free cytosolic Ca^2+^ ions in salt stressed plants. Thus, more research is required to point out the identity of such cytosolic Na^+^ sensor [111].

## 3. Signal Transduction

During salt stress, plants transduce the early stress signals to different cellular machinery called signal transduction. In general, signal transduction starts right after stress sensing, followed by the synthesis of secondary signaling molecules, such as Ca^2+^ and reactive oxygen species (ROS) (Figure 1). The production of secondary signaling molecules modulates the cytosolic Ca^2+^ concentration that binds to different protein kinases, such as calmodulins (CaMs)/CaM-like (CML), calcium-dependent protein kinases (CDPKs), calcineurin B-like interacting protein kinases (CIPKs), and MAPKs. As these protein kinases lack enzymatic activity, they catalyze protein phosphorylation via a Ca^2+^-dependent manner, resulting in protein conformational change. Thus, protein phosphorylation cascades mainly depend on the cytosolic Ca^2+^ concentration [112,113].

### 3.1. CaM/CML

CaM/CML proteins are important Ca^2+^ transducers in plant responses to abiotic stress [114,115]. In rice, five CaM-encoding genes—namely, *OsCam1-1*, *OsCam1-2*, *OsCam1-3*, *OsCam2*, and *OsCam3*—were identified [10]. Among these, *OsCam1-1* is highly activated during salt stress. Yuenyong et al. [116] reported that the rice plants overexpressing *OsCam1-1* affected differential expression of genes involved in signaling, hormone-mediated regulation, transcription, lipid metabolism, carbohydrate metabolism, photosynthesis, glycolysis, tricarboxylic acid cycle, and glyoxylate cycle during salt stress. This further suggests that a complex network of downstream cellular processes is involved in the CaM signal transduction pathway. CaM binds with other proteins and interacts with other signaling cascades, such as plant hormone signaling, during stress conditions. For instance, it binds either with MAPK or mitogen-activated protein kinase phosphatase (MKP) to regulate the MAPK cascades [117]. Recently, six novel proteins—namely, *OsLRK5a*, *OsDCNL2*, *OsWD40-139*, *OsGDH1*, *OsCIP*, and *OsERD2*—were identified as targets of *OsCML16* in responses to salt stress through yeast hybridization and bimolecular fluorescence complementation assay. These target genes are involved in plant hormone signaling processes, including auxin and ABA [118]. Interestingly, both *OsCaM1* and *OsCML16* could bind with *OsERD2* and thus could transduce Ca^2+^ via both CaM and CML proteins [118]. Although the functional role of *OsERD2* in response to salt stress is still unknown, it is speculated that it plays a vital role in programmed cell death during innate immunity, similar with *AtERD2* [119].

### 3.2. CDPK

CDPKs mediate downstream components of the Ca^2+^ signaling cascades by directly binding Ca^2+^ to CaM-like domain. In rice, a total of 29 CDPK genes have been identified [120]. Four rice CDPK genes—namely, *OsCPK4*, *OsCDPK7*, *OsCPK12*, and *OsCPK21*—were functionally validated and act as positive regulators of salt tolerance (Table 1). Overexpression of rice CDPKs upregulate expression of genes involved in lipid metabolism and the active oxygen detoxification system. For instance, overexpression of *OsCPK4* upregulated the genes involved in oxidative stress and redox regulation [11]. Similarly, transgenic rice plants overexpressing *OsCPK12* significantly enhanced the expression of genes encoding reactive oxygen species (ROS) scavenging enzymes, such as *OsAPx2* and *OsAPx8* [14]. *OsCDPK7* positively regulates salt tolerance by regulating salt-stress responsive gene, *rab16A* [12,13]. Meanwhile, *OsCPK21* enhances salt tolerance via regulation of ABA- and salt stress-inducible genes, such as *Rab21*, *OsNAC6*, *OsLEA3*, *OsP5CS*, *OsNHX1*, and *OsSOS1* [15]. Further study revealed that *OsCPK21* regulates salt tolerance by phosphorylating *OsGF14e*/*Os14-3-3* at the Tyr_138_ [16]. This was the first evidence of 14-3-3 protein-associated phosphorylation of CDPK in rice. Despite intensive work in studying the role of CDPKs in regulation of salt tolerance in rice, their role in different signaling cascades needs to be elucidated. 

### 3.3. Calcineurin B-Like Protein (CBL)/CIPK

CBLs are plant-specific Ca^2+^ sensors that bind with CIPKs to relay perceived Ca^2+^ signal, thereby inducing downstream gene regulation for abiotic stress. The *SOS3–SOS2* complex is the first evidence of CBL–CIPK interaction in plant responses to salt stress [121]. Homologues of *SOS2* and *SOS3* in rice, the *OsCIPK24* and *OsCBL4*, have been cloned, which suggests that the SOS pathway also operates in rice responses to salt stress [122]. Further study revealed that *OsCIPK24*/*OsSOS2*, *OsCBL4*/*OsSOS3*, and *OsSOS1* were highly upregulated in salt-tolerant rice cultivars when subjected to salt stress [123]. This suggests that the rice CBL4–CIPK24 complex, together with the Ca^2+^ signal, regulates ion homeostasis similar to *Arabidopsis.* Therefore, the SOS pathway is conserved in both dicots and monocots. Many other CBL and CIPK genes are involved in rice responses to salt stress based on transcriptome analysis [124,125]. However, only *OsCIPK15* and *OsCIPK31* have been functionally validated for their role in salt tolerance. Transgenic rice plants overexpressing *OsCIPK15* showed enhanced salt tolerance with higher free proline and soluble sugar concentration [17]. Similarly, *OsCIPK31* acts as a positive regulator of salt tolerance wherein the loss-of-function mutant *oscipk31*:*Ds* exhibited hypersensitive phenotype under saline condition [18].

### 3.4. MAPK

MAPK is considered the last component of the protein phosphorylation cascade in transducing Ca^2+^ ions in response to environmental stimulus. The MAPK signaling pathway activates different transcription factors (TFs) involved in the production and scavenging of ROS [126]. Three classes of MAPKs are found in plants; namely, MAPK kinase kinase (MKKK), MAPK kinase (MKK), and MAPK [127,128]. Rice has 15 MAPKs, 8 MKKs, and 75 MKKKs, of which a few are involved in salt stress response (Table 1) [129,130,131]. Overexpression and gene silencing validated the role of *OsMAPK5* as a positive regulator of salt tolerance [19]. Further study showed that *OsMAPK5* phosphorylates *SERF1*, a regulator of ROS signaling during initial response to salt stress [34]. Thus, *OsMAPK5* plays an essential role in the ROS signaling pathway. In contrast, *OsMAPK33* acts as a negative regulator and alters the expression of genes involved in Na^+^ transport [20]. *OsMAPKKK63* also acts as a negative regulator of salt tolerance and interacts with *OsMKK1* and *OsMKK6* [23]. Both *OsMKK1* and *OsMKK6* are known mediators of rice responses to salt stress. Overexpression of *OsMKK6* enhances salt tolerance by inducing *MAPK* substrate phosphorylation [22]. Similarly, *OsMKK1* acts as a positive regulator with highly upregulated transcripts under saline conditions [21]. Moreover, yeast hybridization and in-vivo/vitro kinase assays revealed that *OsMPK4* is the downstream target of *OsMKK1*. *OsMPK4* is involved in the wounding signaling pathway in rice [132]. However, its functional role in salt tolerance is not well characterized. 

## 4. Transcriptional Regulation

In the past centuries, numerous proteins were reported to play an important role in salt tolerance. Transcriptomic tools have further subdivided these proteins into two major classes, the functional and regulatory proteins. Functional proteins are those that directly function in protecting the plants from stress. These include ion transporters, antioxidant proteins, osmolytes, water channel proteins, heat shock proteins, and late embryogenesis abundant (LEA) proteins. On the contrary, regulatory proteins, such as transcription factors (TFs), are involved in regulating the complex network of signal transduction [133,134,135,136].

**Figure 2:**
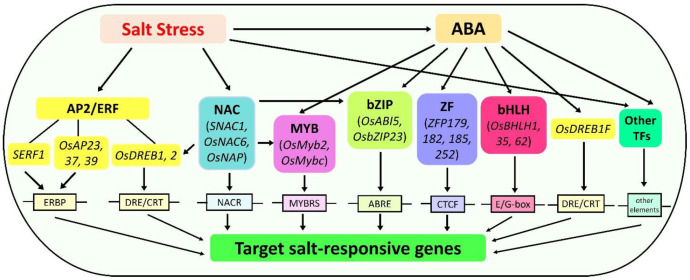
Transcriptional regulation involved in activating salt stress-responsive genes in rice. The transcriptional regulation occurs via abscisic acid (ABA)-dependent and -independent pathway, whereby transcription factors (TFs) bind with their corresponding *cis*-regulatory element. The APETALA2/ethylene responsive factor (AP2/ERF) and NAC (NAM, ATAF, and CUC) TFs operate in an ABA-independent pathway. NAC TFs regulate other TFs, such as dehydration responsive element-binding (DREB), myeloblastosis (MYB), and basic leucine-zipper (bZIP). The MYB, bZIP, zinc finger (ZF), basic-helix-loop-helix (bHLH), DREB, and other TFs are involved in the ABA-dependent pathway.

TFs are key proteins that bind with *cis*-elements in the promoter of target genes, thereby modulating the rate of gene expression in the downstream signaling cascades in response to different environmental cues. A large number of TFs have been identified in rice, with 2025 TFs in *Oryza sativa spp. indica* and 2384 in *spp. japonica* [137]. In recent years, many TFs along with their interacting proteins have been implicated in rice responses to salt stress and regulate a series of signaling pathways (Table 1). Most of these are members of APETALA2/ethylene responsive-factor (AP2/ERF), NAC (NAM, ATAF, and CUC) proteins, myeloblastosis (MYB), basic leucine-zipper (bZIP) type proteins, zinc finger (ZF) and basic helix-loop-helix (bHLH) TFs that regulate many salt stress-responsive genes either through an ABA-dependent or -independent manner (Figure 2). Thus, understanding how TFs, along with their interacting proteins, regulate a network of signaling pathways and their downstream genes is crucial in elucidating the salt tolerance mechanisms of rice.

### 4.1. APETALA2/Ethylene Responsive Factor (AP2/ERF) Regulation

AP2/ERF-type TFs are characterized by the presence of an AP2 DNA-binding domain of approximately 60 amino acids. In rice, at least 163 AP2/ERF TFs have been identified. This TF family is further subdivided into four subfamilies: the AP2, dehydration responsive element-binding (DREB), ERF, and related to ABI3 and VP1 (RAV) proteins [138]. Among these, DREB is widely involved in rice responses to salt stress, though a few AP2-, ERF-, and RAV-type TFs regulate salt tolerance (Table 1). 

DREB binds to the dehydration-responsive element/c-repeat (DRE/CRT) *cis*-elements in the promoter region of stress-responsive genes. DREBs have been isolated in several crops, and their overexpression enhances tolerance to different abiotic stresses, including salinity [139]. Rice DREB1 genes enhance salinity tolerance by regulating osmoprotection, as evident in rice and *Arabidopsis* DREB1 overexpression plants [25,26,140,141]. For instance, *OsDREB1A* targets two dehydrin genes [24]. Dehydrins protect plasma membrane from damage during drought- or salt-induced osmotic stress [142]. Moreover, the level of proline and soluble sugars, which are important for osmotic adjustment, significantly increased in DREB1 overexpression plants [140,143]. DREB genes mainly work in the ABA-independent pathway; however, some also participate in the ABA-dependent pathway, as exemplified by *OsDREB1F*. Transcript profiling in *OsDREB1F* overexpression lines showed expression of ABA-dependent genes, *rd29B* and *RAB18* [26]. DREB2-type genes also act as positive regulators of salt tolerance. Overexpression of *OsDREB2A* and *OsDREB2B* in both rice and *Arabidopsis* improved salt tolerance [24,27,28,29]. Another DREB gene, *OsDREB6*, classified as an A-6 type of DREB TF positively regulates salt tolerance. Transgenic rice plants overexpressing *OsDREB6* showed high levels of proline, soluble sugars, and catalase. Conversely, the levels of these enzymes were significantly reduced in RNAi plants [33]. This suggests that DREB genes mainly enhance salt tolerance by regulating genes responsible for osmoprotection and antioxidation. Similar to DREB, other TFs in the AP2/ERF family enhance salt tolerance by regulating several downstream genes involved in osmotic stress and antioxidant defense system. For instance, *SERF1* gene regulates ROS-dependent signaling as an initial response to salt stress [34]. Recently, Wang et al. [32] demonstrated that *OsSTAP1*, an AP2/ERF-type TF, positively regulates salt tolerance by activating genes encoding antioxidant enzymes (*OsPOD1, OsPOD72, GSTT3*) and aquaporin gene (*NIP2-1*). Unlike most of AP2/ERF-type TFs, *OsERF922* and *OsAP23* act as negative regulators and downregulate the expression of defense-related genes [30,35].

### 4.2. NAC Regulation

NAC proteins are a plant-specific gene family that regulate both ABA-independent and ABA-dependent inducible genes [144]. Several studies have been carried out to understand the role of rice NAC genes in response to abiotic stimulus, including salinity. Most functionally characterized rice NAC proteins act as positive regulators of salt tolerance (Table 1). *SNAC1*, the first stress-related NAC type TF characterized in rice, enhances both drought and salt tolerance [48]. Transcriptome analysis of transgenic plants overexpressing NAC proteins showed upregulation of many stress-inducible genes. For instance, *OsNAC2*, *OsNAC5*, *ONAC022*, and *ONAC106* target *OsLEA3* [38,41,43,44]; *OsNAP* targets several stress-related genes, including *OsPP2C06*/*OsABI2*, *OsPP2C09*, *OsPP2C68*, and *OsSalT* [37]; and *OsNAC2* targets genes involved in osmoprotection (*OsP5CS1*), antioxidation (*OsCOX11*), K^+^-efflux channel genes (*OsGORK* and *OsSKOR*), and ABA-inducible genes (*OsNCED1* and *OsNCED3*) [42,43]. NAC TFs also regulate other stress-related TFs. For instance, *OsNAP* induces the expression of *OsDREB1A* and *OsMYB2* [37]. *ONAC106* binds with the promoter of *OsNAC5*, *OsDREB2A*, and *OsbZIP23* TF genes [41]. Similarly, *ONAC022* targets *OsDREB2a* and *OsbZIP23* (Hong et al. 2016).

### 4.3. MYB Regulation

MYB proteins are one of the richest TF families in plants, representing at least 155 genes in rice. It is considered as an active player in plant development, secondary metabolism, cell differentiation, organ morphogenesis, and response to both biotic and abiotic stresses [145,146]. These TFs mainly participate in the ABA-dependent pathway, upregulating a number of stress-responsive genes. For example, expression of *OsMPS*, an R2R3 type MYB TF, is significantly induced by ABA and regulates several expansin and glucanase genes [54]. Transcriptome analysis of transgenic rice plants overexpressing *OsMYB48-1* upregulates ABA biosynthesis genes (*OsNCED4* and *OsNCED5*), early signaling genes (*OsPP2C68* and *OSRK1*), and late responsive genes (*RAB21*, *OsLEA3*, *RAB16C*, and *RAB16D*) [53]. Similarly, *OsMYB2* targets *OsLEA3* and *OsRab16A* [51]. MYB TFs also regulate the expression of some transporter genes. For example, *OsMYBc* binds with the AAANATNY motif in the promoter of *OsHKT1;1*, thereby upregulating its expression [56]. Other rice MYB TFs involved in the regulation of salt tolerance are presented in Table 1.

### 4.4. bZIP Regulation

bZIP TFs are composed of a highly conserved basic region and a leucine zipper domain of about 60 to 80 amino acids in length. Several rice bZIP TFs are involved in transcriptional activation of several stress-responsive genes, most of which participate in the ABA-dependent pathway (Table 1). Overexpression of *OsbZIP71* upregulates several genes that encode ion antiporters (*OsCLC-1*, *OsNHX1*, *OsHKT6* and *OsVHA-B*) and ROS scavenging (*OsCAT*). Interestingly, *OsbZIP71* directly binds to the promoter of *OsNHX1*, an Na^+^/H^+^ antiporter gene involved in vacuolar compartmentation of Na^+^ ions [60]. *OsbZIP23* acts as a key player in salt tolerance by upregulating osmotic stress-inducible genes, such as dehydrins and LEA proteins [59]. *OsHBP1b*, also categorized under the bZIP TF family, could enhance salt tolerance by activating the genes involved in antioxidant defense system [61]. It is worth noting that *OsHBP1b* is localized within the *Saltol* quantitative trait locus (QTL) region, hence an important salt tolerance gene. Moreover, comparative transcript profiling showed that *OsHBP1b* is highly expressed in popular salt-tolerant rice cultivar Pokkali [147]. Meanwhile, *OsABI5* acts as a negative regulator changing the expression of many salt stress-responsive genes. *OsABI5* significantly downregulates the expression of *OsHKT1;5*/*SKC1* and upregulates *SalT* gene [58]. Transcriptomic analysis showed that many other bZIP TFs play an important role in rice responses to salt stress. However, their regulatory roles have not been functionally studied. Taken together, bZIP TFs mainly regulate salt tolerance via the active oxygen detoxification and ion homeostasis pathways. 

### 4.5. ZF Regulation

ZF proteins are comprised of conserved motifs with cystine (Cys) and histidine (His) residues. These motifs are classified according to the number and order of Cys and His. [148]. Several studies have shown their function in transcriptional activation of several biological processes involved in plant responses to environmental stimulus. Under salt stress conditions, ZF TFs regulate the expression of genes associated with ROS scavenging via ABA-independent and ABA-dependent pathways to reduce oxidative damage. The *ZFP179*, *ZFP182*, and *ZFP252* act as positive regulators of salt tolerance. These ZF TFs transcriptionally activate the *OsDREB1A*, *OsLEA3*, *OsPC5CS,* and *OsProT* genes that are involved in the synthesis of osmolytes, such as proline and soluble sugars [64,65,67]. Conversely, drought and salt tolerance *(DST)* and *ZFP185* act as negative regulators and downregulate several ABA-inducible genes, such as *Prx24* [62,66]. Meanwhile, *OsLOL5*, an LSD1-like-type ZF is involved in transcriptional activation of *OsAPX2*, *OsCAT*, and *OsCu/Zn-SOD* [68]. Thus, ZF TFs play an essential role in the ROS signaling pathway. 

### 4.6. bHLH Regulation

bHLH TFs widely exist in eukaryotic organisms and contain a conserved basic region and a helix-loop-helix (HLH) domain [149]. These TFs play an essential role in several abiotic stress tolerance, wherein several bHLH TF genes have been functionally validated. Concerning salt tolerance, only a few were functionally validated. Three previously reported bHLH TFs enhance salt tolerance in rice by activating ion transporters genes. For instance, *OsbHLH035* enhances salt tolerance by activating Na^+^ transporter genes, *OsHKT1;3* and *OsHKT1;5/SKC1*, which are involved in Na^+^ loading and unloading [70]. *OrbHLH001* enhances Na^+^ efflux and K^+^ influx under salt stress by activating *OsAKT1* [69]. Meanwhile, *OsbHLH062* acts as transcriptional activator of *OsHAK21* in response to salt stress [150]. The bHLH TFs therefore regulate salt tolerance via the ion homeostasis pathway. Moreover, these TFs activate gene expression through their interaction with the specific E-box motif in the promoter of the target gene [69,141,151]. 

### 4.7. Other TFs Involved in Salt Tolerance

In addition to the TFs previously discussed, many other TF families play an essential role in reprogramming transcriptome during salt stress. The homeodomain-leucine zipper (HD-Zip) TF family is also important for salt tolerance, such as *Oshox22* and *OsTF1L*. *Oshox22* acts a negative regulator of salt tolerance and is upstream to *OsbZIP23* [71]. *OsTF1L* positively regulates salt tolerance mainly by regulating genes involved in stomatal closure and lignin biosynthesis [72].

Apart from *OsbZIP71*, previously discussed, several TFs belonging to different families regulate the expression of the *OsNHX1* transporter gene. The *OsNIN-like4* and *OsPCF2*, a nodule inception (NIN) and teosinte branched 1/cycloidea/proliferating cell (TCP) proteins, respectively, act as transcriptional activators of *OsNHX1*. Conversely, *OsCPP5* and *OsNIN-like2* act as repressors [152]. *OsMADS25*, a MADS-box TF gene, acts as positive regulator by upregulating the expression of genes involved in the ROS detoxification system [73]. Meanwhile, the WRKY-type TF, *OsWRKY45*, negatively regulates salt tolerance [74].

## 5. Salt Tolerance Adaptive Mechanisms

**Figure 3:**
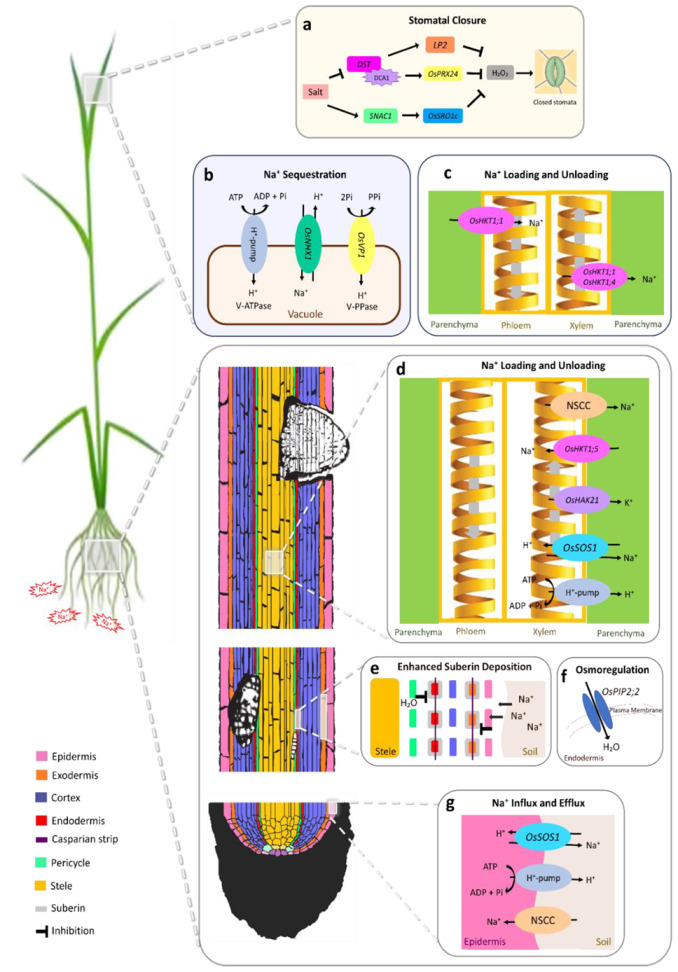
Rice salt tolerance adaptive mechanisms. In the leaf, (**a**) stomatal closure mediated either by *DST* or *SNAC1* is the initial response of rice to salinity. Salt stress downregulates *DST* which interacts with *DCA1* and activates *OsPrx24* and *LP2*. Conversely, *SNAC1* is upregulated, activating the *OsSRO1c*. These downstream genes mediate stomatal closure via H_2_O_2_ inhibition. (**b**,**c**) Na^+^ content in the leaf cytoplasm is controlled by vacuolar sequestration, xylem unloading, and phloem loading. Excess Na^+^ is sequestered into the vacuole via *OsNHX1* coupled with H^+^-pump and *OsVP1*, a vacuolar-type H^+^-pyrophosphatase encoding gene. Na^+^ unloading at the xylem and Na^+^ loading at the phloem are mediated by *OsHKT1;4* and *OsHKT1;1*, respectively. In the root, (**d**) Na^+^ is loaded at the xylem through nonselective cation channel (NSCC) and *OsSOS1* coupled with H^+^-pump. Conversely, *OsHKT1;5* unloads the Na^+^ ions from the xylem and shuttles them back to the parenchyma cells. Apart from Na^+^, K^+^ influx occurs mediated by *OsHAK21*, thereby increasing the K^+^/Na^+^ ratio. (**e**) Enhanced suberin deposition in the root exodermis and endodermis also inhibits Na^+^ influx to the stele. Similarly, it blocks water transport out of the stele. (**f**) The plasma membrane-bound *OsPIP2;2* gene increases hydraulic conductivity in the root endodermis, allowing water uptake. (**g**) Na^+^ enters the root epidermis via NSCC and is shuttled back to the external medium via the *OsSOS1* coupled with H^+^-pump.

Several adaptive mechanisms have been observed in plant responses to salt stress. In rice, osmoregulation, stomatal closure, and development of antioxidant enzymes are the immediate responses during salt stress. This is later followed by Na^+^ exclusion and sequestration upon uptake of toxic Na^+^ ions. The tissue specific localization of genes that regulate salt tolerance adaptive traits in rice is presented in Figure 3.

### 5.1. Osmoprotection and Osmoregulation

Cell dehydration due to low osmotic potential of soil water is the immediate effect of salt stress. Under such a situation, plants (1) synthesize compatible solutes, known as osmolytes, to maintain cell turgor and (2) activate water channel aquaporins that regulate water uptake. 

#### 5.1.1. Osmolytes

Several osmolytes, such as trehalose and glycine betaine (GB), have been proven effective in preventing cellular dehydration during salt stress [153]. Thus, exogenous application of osmolytes has been utilized to enhance salt tolerance in rice [154,155,156,157]. However, very few studies have been conducted to characterize osmolyte encoding genes for their role in salt tolerance. 

The two key enzymes in trehalose biosynthesis, trehalose-6-phosphate phosphatase (TPP) and trehalose-6-phosphate synthase (TPS), are involved in rice responses to salinity. The *OsTPP1*, *OsTPS1*, and *OsTPS8* positively regulate salt tolerance by increasing the accumulation of trehalose and proline in rice overexpression plants [77,78,79]. 

GB is also an important osmolyte under salt stress that prevents lipid peroxidation [158]. Additionally, accumulation of high GB enhances photosynthetic activity [159]. The *OsBADH1*, a major gene involved in converting betaine aldehyde to GB, plays an important role in salt tolerance. This gene prevents oxidative damage, protects chlorophyll degradation, and ultimately prevents leaf senescence during salt stress [75]. Moreover, RNAi-directed knockdown of *OsBADH1* enhances the production of ROS, causing lipid peroxidation [76]. Thus, the gene acts as a positive regulator of salt tolerance.

#### 5.1.2. Water Channel Aquaporins

Plant aquaporins also play a significant role in osmoregulation. Aquaporins are membrane-localized channels that are mainly involved in water transport and homeostasis [160,161]. Rice has 33 aquaporins, few of which regulate root hydraulic conductivity under saline condition [162]. Overexpression of *OsPIP1;1* and *OsPIP2;2*, plasma membrane intrinsic proteins (PIPs) family genes in *Arabidopsis*, enhanced salt tolerance by maintaining water homeostasis [80]. Likewise, rice overexpressing *OsPIP1;1* increased root hydraulic conductivity under salt stress [81]. Rice aquaporins might be coordinately orchestrated in maintaining water homeostasis based on their organ-specific transcript expression. Transcript of *OsPIP2* genes were highly expressed in the roots; thus, it could be the predominant gene regulating water uptake in the roots (Figure 3f). Conversely, the *OsPIP1* gene transcript was the highest in the leaves, suggesting its role in leaf water transport [80]. Apart from the PIP genes, several tonoplast intrinsic protein (TIP) genes also play an important role in salt-induced osmotic stress [163]. 

### 5.2. Stomatal Closure

Stomatal closure is the initial response of plants under salinity and is controlled by both ABA and ROS signaling [164]. *DST* mainly regulates salt tolerance via stomatal closure under salt-induced osmotic stress. Further study revealed that a leucine-rich repeat (LRR)-RLK gene, *LP2*, required for stomatal closure is downstream to *DST* [82]. Interestingly, *DST* interacts with *DST co-activator 1* (*DCA1*) and regulates the expression of *OsPrx24*, a gene encoding H_2_O_2_ scavenger [165]. Meanwhile, *OsSRO1c*, expressed in the guard cells and a downstream gene target of *SNAC1* TF, also regulates stomatal closure under both drought- and salt-induced osmotic stress (Figure 3a). Overexpression of *OsSRO1c* in rice plants showed enhanced stomatal closure and maintained H_2_O_2_ homeostasis under salt stress. Conversely, knockdown mutants showed high sensitivity to osmotic stress [83].

### 5.3. Antioxidation

ROS synthesis is important in different signaling and physiological processes. However, overproduction of ROS is deleterious to different cellular components, such as proteins, nucleic acids, and membrane lipids. Thus, plants synthesize ROS scavenging enzymes to maintain redox homeostasis [126,166]. In this section, we discuss genes encoding ROS scavenging enzymes that are involved in rice responses to salt stress.

#### 5.3.1. Superoxide Dismutase (SOD)

SODs catalyze the first step in the reactive-oxygen scavenging system by dismutation of the highly toxic O_2_^−^ to H_2_O_2_. Thus, it is considered the most effective intracellular antioxidant enzyme. Rice has three distinct types of SOD isoforms that are differentiated according to the metals they contain, either Cu/Zn, Mn, or Fe. The activity of these SODs is associated with specific subcellular localization: Mn-SOD is located in both mitochondria and peroxisomes; Fe-SOD is located in the chloroplasts; and Cu/Zn-SOD is located in the chloroplasts, cytosol, and peroxisome [167]. The expression of genes encoding these SOD isoforms is highly influenced by salt stress and is activated by ZF-type TFs, as discussed in Section 4.5. Mishra et al. [168] reported that the increase in SOD activity of salt-tolerant rice cultivar CSR27 exposed to salinity was directly related to the upregulation of Cu/Zn-SOD encoding genes. Similar results were reported by Rossatto et al. [169], who observed upregulation of five Cu/Zn isoforms (*OsCu/Zn-SOD*, *OsCu/Zn-SOD2*, *OsCu/Zn-SOD3*, *OsCu/Zn-SOD4*, *OsCu/Zn-SODCc1*) under salt stress. Moreover, the rice plants overexpressing chloroplastic *OsCu/Zn-SOD* showed less salt-induced oxidative damage owing to higher ROS detoxification [84]. Upregulation of *OsMn-SOD* was also observed in rice subjected to salt stress. Tanaka et al. [85] reported that overexpression of *OsMn-SOD* in the chloroplasts significantly increased SOD activity and therefore enhanced salt tolerance. Similar results were observed in other plants such as wheat and tall fescue [170,171]. Conversely, salinity downregulates the expression of *OsFe-SOD*, thereby reducing the total SOD activity [172]. This suggests that Cu/Zn-SOD and Mn-SOD isoforms play vital roles in ROS detoxification system during stress condition.

#### 5.3.2. Catalase (CAT)

CATs are strong antioxidant enzymes primarily located in the peroxisome that directly catalyze the conversion of H_2_O_2_ to water and oxygen [173,174]. Thus, it is indispensable in the ROS detoxification system. Cloning and characterization of the rice CAT genes predicted three isoforms; namely, *OsCatA*, *OsCatB*, and *OsCatC* [175]. These genes are transcriptionally activated by bZIP- and ZF-type TFs, as described in Section 4.4 and Section 4.5. RLK is also involved in transcriptional activation of CAT genes. For instance, the *salt tolerance receptor-like cytoplasmic kinase 1* (*STRK1*) activates *OsCatC* via phosphorylation at the Tyr_120_ [176]. Several environmental factors, such as salinity, affect expression of CAT genes. Under saline condition, elevated levels of CAT activity were observed in salt-tolerant rice cultivars [177]. Interestingly, high *OsCatB* and *OsCatC* activity was observed in salt-tolerant plants grown under salt stress [178]. A similar result was reported by Wutipraditkul et al. [179], who observed an inhibitory effect of *OsCatC* in response to salt stress. 

#### 5.3.3. Ascorbate Peroxidase (APX)

APXs, which exist in compartment-specific isoforms, have a higher affinity for H_2_O_2_ than CATs. Thus, they detoxify even at very low H_2_O_2_ concentrations. Rice has eight APX encoding genes: the cytosolic isoforms *OsAPx1* and *OsAPx2*; the peroxisome isoforms *OsAPx3* and *OsAPx4*; and the chloroplastic isoforms *OsAPx5*, *OsAPx6*, *OsAPx7*, and *OsAPx8.* The *OsAPx6* isoform is also localized in the mitochondria [180]. All these APX encoding genes, except *OsAPx3* and *OsAPx5*, were upregulated in rice under salt stress [178,181]. Overexpression of *OsAPx2* showed very high APX activity, thereby enhancing salt tolerance in rice [87]. Likewise, overexpression of either *OsAPx1* or *OsAPx2* exhibited high tolerance to salt stress in *Arabidopsis*; however, *OsAPx2* confers better tolerance than *OsAPx1* [86]. Further study revealed that silencing both *OsAPx1* and *OsAPx2* genes in rice resulted in normal growth and development under salt stress. This is attributed to the upregulation of CAT and APX genes [182,183]. Thus, deficiency of APXs is compensated by other antioxidant enzymes.

#### 5.3.4. Glutathione Reductase (GR)

GRs are flavoprotein oxidoreductases and are important components of the ascorbate (AsA)-glutathione (GSH) cycle [184]. Rice has three GR isoforms: *OsGR1,* located in the cytosol; and *OsGR2* and *OsGR3,* located in both mitochondria and chloroplasts [185]. These rice GRs have been implicated for their role in different abiotic stimuli, including salinity. Salt stress enhances the expression of *OsGR2* and *OsGR3* via the ROS detoxification system [185,186,187]. Further study demonstrated that *OsGR3*, primarily expressed in the roots, positively regulates salt tolerance [88].

#### 5.3.5. Thioredoxin (TRX) and Glutaredoxin (GRX)

TRXs and glutaredoxin (GRX) are key players in redox regulation, therefore considered as redox-sensing compounds. TRX are reduced by TRX reductase, whereas GRX utilizes glutathione as a cofactor in the ROS scavenging system [188]. The rice genome has 30 and 48 genes encoding TRX and GRX, respectively. However, only a few have been functionally validated for their role in salinity tolerance [189,190]. For instance, *OsTRXh1*/*OsTRX23* negatively regulates salt tolerance. RNAi-directed knockdown of this gene resulted in salt sensitivity, possibly due to its inhibitory activity on stress-activated MAPKs [89,191]. *OsTRXh1*/*OsTRX23* also inhibits the kinase activity of *OsMPK3* and *OsMPK6* [192]. Meanwhile, *OsGRX8* and *OsGRX20* positively regulate salt tolerance by restraining the accumulation of O_2_^-^ radicals [90,91]. 

### 5.4. Na^+^ Exclusion and Sequestration

Na^+^ ions are the major toxic element taken up by the plant during salt stress. Maintaining low levels of toxic Na^+^ ions in the cytosol, either through Na^+^ exclusion or sequestration, is the most effective strategy to avert the deleterious effects of salinity. Glycophytes, such as rice, exclude Na^+^ from the shoot either by (i) Na^+^ efflux from roots to the rhizosphere, (ii) Na^+^ loading and unloading at the xylem, or (iii) vacuolar Na^+^ compartmentation.

#### 5.4.1. Na^+^ Efflux

The efflux of Na^+^ ions across the root plasma membrane into the external medium is poorly understood. Nevertheless, it is central to the Na^+^ exclusion mechanisms in plants [1]. To date, only *SOS1*, coupled with H^+^-ATPases, is the major Na^+^ efflux transporter that has been genetically characterized in plants [110,193]. The rice *SOS1* ortholog (*OsSOS1*) is expressed in epidermal cells at the root cap and in cells around the xylem similar with *Arabidopsis AtSOS1* [194]. The *OsSOS1* activity, catalyzed by Na^+^/H^+^ exchange at the plasma membrane, could suppress Na^+^ sensitivity of yeast mutant lacking the Na^+^ efflux system, thus reducing the net cellular Na^+^ concentration. Similarly, *OsSOS1* complementation in *Arabidopsis* mutant *sos1-1* reduced growth defect in both saline and non-saline conditions [122]. Further study demonstrated that rice *sos1* loss-of-function mutant displayed very high root Na^+^ uptake and impaired Na^+^ loading into the xylem [95]. Thus, *OsSOS1* plays a critical role in Na^+^ efflux from root epidermal cells to the rhizosphere.

#### 5.4.2. Na^+^ Loading and Unloading

Na^+^ loading and unloading at the xylem is regulated by high-affinity K^+^ transporters (HKTs). HKTs are among the most well characterized Na^+^ and/or K^+^ plant transporters identified in several plants and play a central role in salt tolerance [195,196]. Two HKTs are highlighted in a proposed two-staged Na^+^ exclusion mechanism, whereby the (i) *OsHKT1;5*/*SKC1* mediates root-to-shoot Na^+^ transfer and (ii) *OsHKT1;4* mediates leaf sheath-to-blade Na^+^ transfer. The Na^+^ ions entering the root xylem via nonselective cation channel (NSCC) are shuttled back to the parenchyma via *OsHKT1;5*/*SKC1* (Figure 3d). Meanwhile, *OsHKT1;4* not only functions in Na^+^ unloading to the leaf sheath, but also to the stem during the reproductive stage [197]. Further study revealed that *OsHKT1;4* is involved in leaf Na^+^ exclusion via Na^+^ unloading at the xylem (Figure 3c). The mutant line overexpressing *OsHKT1;4* showed salt sensitivity owing to very high root Na^+^ uptake [93]. Thus, a coordinated balance in root and shoot Na^+^ exclusion is essential to achieve salt tolerance. Another HKT1 gene, *OsHKT1;1*, transcriptionally activated by *OsMYBc* as previously discussed, is also reported to regulate Na^+^ exclusion, possibly through both Na^+^ unloading from the xylem and Na^+^ loading into the phloem (Figure 3c). The Na^+^ loaded into the phloem is hypothesized to be recirculated from shoots to roots or from young leaves to old leaves, thereby reducing salt injury in newly emerging leaf [56]. Moreover, it was demonstrated that *OsHKT1;1* is a positive regulator of salt tolerance that mediates Na^+^ exclusion from the shoot [92]. Recent studies have shown that there are eight and four transcript variations of HKT1 genes with different lengths in *O. sativa spp. indica* and *spp. japonica*, respectively. These eight transcript variations in *O. sativa spp. indica* show different expression levels and transport activities under salt treatment, which suggests the existence of different transport mechanisms [198]. 

#### 5.4.3. Vacuolar Na^+^ Sequestration

Few rice cultivars with high Na^+^ concentrations in the leaves were found to perform well under saline condition. This is mainly due to the active compartmentation of Na^+^ ions into the vacuole, also known as tissue tolerance, mediated by the tonoplast localized Na^+^/H^+^ antiporters (NHX) and energized by a proton motive force (Figure 3b) [193]. This mechanism allows the plant to use Na^+^ ions in maintaining cell turgor, and hence continuous plant growth under salt [199,200]. Additionally, vacuolar Na^+^ sequestration maintains cytosolic alkalinity and vacuolar acidity. Maintaining low vacuolar pH is essential since acidity allows the vacuole to isolate and break down misfolded proteins [201]. This phenomenon was only observed in salt-tolerant rice cultivars, such as Pokkali [111]. 

Four vacuolar NHX genes—namely, *OsNHX1*, *OsNHX2*, *OsNHX3*, and *OsNHX5*— were identified in rice mediating cytosolic Na^+^ sequestration into the vacuole [202]. Further study revealed that overexpression of *OsNHX1* enhanced tissue tolerance and is regulated by *OsbZIP71* TF [60,96,203]. Very high transcripts of these NHX genes in either flag leaf or panicle has also been observed [202]. This suggests their potential role in enhancing salt tolerance at the reproductive stage.

Functional characterization of vacuolar-type H^+^-pyrophophatase (H^+^-PPase) also showed enhanced salt tolerance. H^+^-PPase is the main driving force for Na^+^ transport from the cytoplasm to the vacuole (Figure 3b). Overexpression of H^+^-PPase encoding genes in different plants significantly enhanced salt tolerance [204,205,206]. In rice, overexpression of *OsVP1*, a H^+^-PPase encoding gene, resulted in less serious Na^+^ toxicity under salt stress. Moreover, double overexpression of *OsNHX1* and *OsVP1* conferred better salt tolerance [96]. This is possibly due to the higher electrochemical gradient brought by *OsVP1* overexpression, thereby promoting higher activity of *OsNHX1* (Figure 3b). Interestingly, a similar result has been found in simultaneous expression of *SsNHX1* from *Suaeda salsa* and *AVP1* from *Arabidopsis* in rice [206].

### 5.5. Suberin Deposition

Suberin deposition is essential in blocking apoplastic leakage of Na^+^ ions into the stele, resulting in low concentration of Na^+^ ions that can be transported into the shoot (Figure 3e). In rice, a few studies have reported the role of suberin in salt tolerance. Enhancing suberin in the form of silicon has significantly reduced the root-to-shoot Na^+^ uptake by preventing apoplastic Na^+^ transport across the root [207]. Interestingly, the popular salt-tolerant rice, Pokkali, showed higher suberin deposition compared with the salt-sensitive cultivar IR20 [208]. However, the gene regulatory network involved in suberin deposition and salt tolerance in rice is not well understood. The *OsTPS8*, involved in trehalose biosynthesis, was also reported to enhance salt tolerance, mainly by enhancing suberin deposition [79].

### 5.6. K^+^ Uptake

Cytosolic K^+^ concentration has emerged as an important aspect of a plant’s adaptability to salt stress, wherein high K^+^ concentration directly relates to salt tolerance. Four high-affinity K^+^ transporter (HAK) genes—namely, *OsHAK1*, *OsHAK5*, *OsHAK16*, and *OsHAK21*—play crucial roles in K^+^ homeostasis under stress conditions [97,98,99,100]. Interestingly, differences in spatial expression were observed among these HAK genes. β-glucoronidase (GUS) staining assay showed that *OsHAK1*, *OsHAK5*, and *OsHAK16* were mainly expressed in the root epidermis [97,98,100]. Conversely, *OsHAK21* was mainly expressed in the root xylem parenchyma [99]. Thus, *OsHAK21* is likely the predominant gene mediating K^+^ influx in the xylem (Figure 3d). 

## 6. Conclusions and Perspectives

Soil salinity, apart from drought and flooding, is a serious menace afflicting global rice production. Being the staple crop of half of the world’s population, developing salt-tolerant rice varieties is crucial, requiring a better overview on molecular and physiological responses to salt stress. Rice responds to salinity through different biological processes, starting with salt stress sensing. Sensing is mediated by different sensors. The sensors relay stress signals to secondary messengers that activate protein phosphorylation cascades and finally the transcriptional regulation of stress-responsive genes via abscisic acid (ABA)-independent/ABA-dependent pathways. Rice response to salt stress also involves several signaling components, transcription factors, and functional genes that directly mediate osmoregulation, antioxidation, and ion homeostasis. Despite the characterization of these genes, understanding the molecular mechanism of rice responses to salt stress remains a great challenge. 

Over the last few decades, remarkable progress in understanding the genomics-physiology of salinity tolerance in plants has taken place. Several genes have been identified to confer salt tolerance in rice; however, most were achieved through a reverse genetics approach. Thus, a large number of genes need to be identified via forward genetics. The current understanding of the molecular responses of rice to salt stress from sensing and signaling up to the development of adaptive tolerance mechanisms is still obscure and requires further research. In particular, identification of upstream pathways and the molecular mechanisms involved in salt stress sensing is crucial to clearly disentangle the osmotic and Na^+^ stress responses in rice. To date, only the role of ABA signaling in rice responses to salt stress is widely studied. The crosstalk between signaling pathways and of other hormones, including auxin, gibberellic acid, jasmonic acid, and ethylene, is still not clear and needs further investigation. Studying the epigenetic regulations of salt tolerance in rice is another important field to dissect. Epigenetic mechanisms control the expression of stress-responsive genes in response to internal and environmental cues. Thus, epigenomic variations may provide a useful resource of DNA methylomes that can be used to better understand the complex salt tolerance mechanisms in rice. 

## Figures and Tables

**Table 1:** List of functionally validated candidate genes involved from sensing to development of salt tolerance adaptive mechanisms in rice.

Gene Name	Gene ID	Functional Annotation	Method of Validation	* Regulation Role	References
Osmosensing					
*SIT1*	LOC_Os02g42780	lectin receptor-type protein kinase	Knockdown Overexpression	–	[9]
Signaling					
*OsCam1-1*	LOC_Os03g20370	Calmodulin	Overexpression	+	[10]
*OsCPK4*	LOC_Os02g03410	CAMK_CAMK_like.12—Ca^2+^/calmodulin-dependent protein kinase(CAMK) includes calcium/calmodulin dependent protein kinases	Knockdown Overexpression	+	[11]
*OsCDPK7*	LOC_Os04g49510	CAMK_CAMK_like.27—CAMK includes calcium/calmodulin dependent protein kinases	Overexpression	+	[12,13]
*OsCPK12*	LOC_Os04g47300	CAMK_CAMK_like.26—CAMK includes calcium/calmodulin dependent protein kinases	Overexpression	+	[14]
*OsCPK21*	LOC_Os08g42750	CAMK_CAMK_like.37—CAMK includes calcium/calmodulin dependent protein kinases	Overexpression	+	[15,16]
*OsCIPK15*	LOC_Os11g02240	CAMK_Nim1_like.4—CAMK includes calcium/calmodulin dependent protein kinases	Overexpression	+	[17]
*OsCIPK31*	LOC_Os03g20380	CAMK_Nim1_like.2—CAMK includes calcium/calmodulin dependent protein kinases	Mutant	+	[18]
*OsMAPK5*	LOC_Os03g17700	CGMC_MAPKCGMC_2_ERK.2—CGMC includes CDA, MAPK, GSK3, and CLKC kinases	Knockdown Overexpression	+	[19]
*OsMAPK33*	LOC_Os02g05480	CGMC_MAPKCMGC_2_SLT2y_ERK.1—includes cytidine deaminase (CDA), glycogen synthase kinase 3 (GSK3), mitogen-activated protein kinase (MAPK), and CLKC kinases	Knockdown Overexpression	–	[20]
*OsMKK1*	LOC_Os06g05520	MAPK	Knockdown	+	[21]
*OsMKK6*	LOC_Os01g32660	STE_MEK_ste7_MAP2K.2—STE kinases	Overexpression	+	[22]
*OsMaPKKK63*	LOC_Os01g50370	STE_MEKK_ste11_MAP3K.4—STE kinases	Knockdown	–	[23]
Transcriptional regulation					
*OsDREB1A*	LOC_Os09g35030	Dehydration-responsive element (DRE)--binding protein	Overexpression	+	[24]
*OsDREB1D*	LOC_Os06g06970	DRE--binding protein	Overexpression	+	[25]
*OsDREB1F*	LOC_Os01g73770	DRE--binding protein	Overexpression	+	[26]
*OsDREB2A*	LOC_Os01g07120	APETALA2 (AP2) domain containing protein	Overexpression	+	[27,28]
*OsDREB2B*	LOC_Os05g27930	AP2 domain containing protein	Overexpression	+	[29]
*OsAP23*	LOC_Os03g05590	AP2 domain containing protein	Overexpression	–	[30]
*OsAP37*	LOC_Os01g58420	AP2 domain containing protein	Overexpression	+	[31]
*OsSTAP1*	LOC_Os03g08470	APETALA2/ethylene responsive factor (AP2/ERF)-type transcription factor	Overexpression	+	[32]
*OsDREB6*	LOC_Os09g20350	ERF transcription factor	Knockdown Overexpression	+	[33]
*SERF1*	LOC_Os05g34730	ERF020- transcription factor	Knockdown	+	[34]
*OsERF922*	LOC_Os01g54890	Ethylene-responsive transcription factor 2	Knockdown Overexpression	–	[35]
*OsRAV2*	LOC_Os01g04800	B3 DNA binding domain containing protein	Mutant	+	[36]
*OsNAP*	LOC_Os03g21060	No apical meristem (NAM)protein	Overexpression	+	[37]
*ONAC022*	LOC_Os03g04070	NAM protein	Overexpression	+	[38]
*ONAC045*	LOC_Os11g03370	NAM protein	Overexpression	+	[39]
*ONAC063*	LOC_Os08g33910	NAM protein	Overexpression	+	[40]
*ONAC106*	LOC_Os08g33670	NAM protein	Overexpression	+	[41]
*OsNAC2*	LOC_Os04g38720	NAM protein	Overexpression	+	[42,43]
*OsNAC5*	LOC_Os11g08210	NAM protein	Knockdown Overexpression	+	[44,45]
*OsNAC6/SNAC2*	LOC_Os01g66120	NAM protein	Overexpression	+	[46,47]
*SNAC1*	LOC_Os03g60080	NAM, ATAF and CUC (NAC) domain-containing protein 67	Overexpression	+	[48]
*OsNAC10*	LOC_Os11g03300	NAC domain transcription factor	Overexpression	+	[49]
*OsNAC041*	-	-	Knockdown	+	[50]
*OsMYB2*	LOC_Os03g20090	Myeloblastosis (MYB) family transcription factor	Overexpression	+	[51]
*OsMYB3R-2*	LOC_Os01g62410	MYB family transcription factor	Overexpression	+	[52]
*OsMYB48-1*	LOC_Os01g74410	MYB family transcription factor	Overexpression	+	[53]
*OsMPS*	LOC_Os02g40530	MYB family transcription factor	Overexpression	+	[54]
*OsMYB91*	LOC_Os12g38400	MYB family transcription factor	Knockdown Overexpression	+	[55]
*OsMYBc*	LOC_Os09g12770	Adenosine-thymine (AT) hook motif domain containing protein	Mutant	+	[56]
*OsABF2*	LOC_Os06g10880	Basic leucine-zipper (bZIP) transcription factor	Mutant	+	[57]
*OsABI5*	LOC_Os01g64000	bZIP transcription factor	Overexpression	–	[58]
*OsbZIP23*	LOC_Os02g52780	bZIP transcription factor	Overexpression	+	[59]
*OsbZIP71*	LOC_Os09g13570	CPuORF2—conserved peptide uORF-containing transcript	Knockdown Overexpression	+	[60]
*OsHBP1b*	LOC_Os01g17260	Transcription factor	Overexpression	+	[61]
*DST*	LOC_Os03g57240	ZOS3-19—C2H2 zinc finger (ZF) protein	Mutant	–	[62]
*OsTZF1*	LOC_Os05g10670	ZF CCCH type family protein	Knockdown Overexpression	+	[63]
*ZFP179*	LOC_Os01g62190	ZOS1-15—C2H2 ZF protein	Overexpression	+	[64]
*ZFP182*	LOC_Os03g60560	ZOS3-21—C2H2 ZF protein	Overexpression	+	[65]
*ZFP185*	LOC_Os02g10200	ZF A20 and AN1 domain-containing stress-associated protein	Knockdown Overexpression	–	[66]
*ZFP252*	LOC_Os12g39400	ZOS12-09—C2H2 ZF protein	Knockdown Overexpression	+	[67]
*OsLOL5*	LOC_Os01g42710	LSD1-like-type ZF protein	Overexpression	+	[68]
*OrbHLH001*	LOC_Os01g70310	Inducer of CBF expression 2	Overexpression	+	[69]
*OsbHLH035*	LOC_Os01g06640	Basic helix-loop-helix (bHLH)	Mutant	+	[70]
*Oshox22*	LOC_Os04g45810	Homeobox associated leucine zipper	Mutant Overexpression	–	[71]
*OsTF1L*	LOC_Os08g19590	Homeobox domain containing protein	Knockdown Overexpression	+	[72]
*OsMADS25*	LOC_Os04g23910	MADS-box family gene with MIKCc type-box	Knockdown Overexpression	+	[73]
*OsWRKY45*	LOC_Os05g25770	WRKY45	Knockdown Overexpression	–	[74]
Osmoprotection					
*OsBADH1*	LOC_Os04g39020	Aldehyde dehydrogenase	Knockdown; Overexpression	+	[75,76]
*OsTPP1*	LOC_Os02g44230	CPuORF22—conserved peptide uORF-containing transcript	Overexpression	+	[77]
*OsTPS1*	LOC_Os05g44210	Trehalose-6-phosphate synthase	Overexpression	+	[78]
*OsTPS8*	LOC_Os08g34580	Trehalose-6-phosphate synthase	Mutant	+	[79]
			Overexpression		
Osmoregulation					
*OsPIP1;1*	LOC_Os02g44630	Aquaporin protein	Overexpression	+	[80,81]
*OsPIP2;2*	LOC_Os02g41860	Aquaporin protein	Overexpression	+	[80]
Stomatal Closure					
*LP2*	LOC_Os02g40240	Receptor kinase	Overexpression	+	[82]
*OsSRO1c*	LOC_Os03g12820	ATP8	Mutant Overexpression	+	[83]
Antioxidation					
*OsCu/Zn-SOD*	LOC_Os08g44770	Copper/zinc superoxide dismutase	Overexpression	+	[84]
*OsMn-SOD*	LOC_Os05g25850	Manganese superoxide dismutase	Overexpression	+	[85]
*OsAPx1*	LOC_Os03g17690	Cytosolic Ascorbate Peroxidase encoding gene 1-8	Overexpression	+	[86]
*OsAPx2*	LOC_Os07g49400	Cytosolic Ascorbate Peroxidase encoding gene 4,5,6,8	Knockdown	+	[87]
			Overexpression		
*OsGR3*	LOC_Os10g28000	Glutathione reductase	Knockdown	+	[88]
*OsTRXh1/OsTrx23*	LOC_Os07g08840	Thioredoxin	Knockdown; Overexpression	–	[89]
*OsGRX8*	LOC_Os02g30850	OsGrx_C8—Glutaredoxin subgroup III	Knockdown; Overexpression	+	[90]
*OsGRX20*	LOC_Os08g44400	Glutathione S-transferase	Knockdown; Overexpression	+	[91]
Na^+^ exclusion					
*OsHKT1;1*	LOC_Os04g51820	Na^+^ transporter	Natural variation	+	[92]
*OsHKT1;4*	LOC_Os04g51830	Na+ transporter	Mutant	–	[93]
*OsHKT1;5/SKC1*	LOC_Os01g20160	Na^+^ transporter	Natural variation	+	[94]
*OsSOS1*	LOC_Os12g44360	Sodium/hydrogen exchanger 7	Mutant	+	[95]
Na^+^ compartmentation					
*OsNHX1*	LOC_Os07g47100	transporter, monovalent cation:proton antiporter-2 family	Overexpression	+	[96]
*OsVP1*	LOC_Os01g68370	B3 DNA binding domain containing protein	Overexpression	+	[96]
K^+^ uptake					
*OsHAK1*	LOC_Os04g32920	Potassium transporter	Mutant and overexpression	+	[97]
*OsHAK5*	LOC_Os01g70490	Potassium transporter	Knockdown overexpression	+	[98]
*OsHAK16*	LOC_Os03g37840	Potassium transporter	Overexpression	+	[99]
*OsHAK21*	LOC_Os03g37930	Potassium transporter	Knockdown	+	[100]

* + positive regulation; – negative regulation.

## Data Availability

Not applicable.

## Abbreviations

AP2/ERF	APETALA2/ethylene responsive factor
APX AT	Ascorbate peroxidase Adenosine-thymine
bHLH	Basic-helix-loop-helix
Bzip CAMK	Basic leucine-zipper Calcium/calmodulin-dependent protein kinase
CAT	Catalase
CBL CDA	Calcineurin B-like protein Cytidine deaminase
CDPK	Calcium-dependent protein kinase
CIPK	CBL-interacting protein kinase
CaM	Calmodulin
CML	Calmodulin-like protein
CPP	Cysteine-rich poly comb-like protein
DRE/CRT	Dehydration-responsive element/c-repeat
DST	Drought and salt tolerance
DCA1	DST co-activator 1
GB	Glycine betaine
GR GUS	Glutathione reductase β-glucoronidase
HAK	High-affinity potassium transporter
HD-Zip	Homeodomain-leucine zipper
HKT	High-affinity K^+^ transporter
HpT	Histidine-containing phosphotransfer protein
LEA	Late embryogenesis abundant
LRR-RLK	Leucine-rich repeat-receptor-like kinase
MAPK	Mitogen-activated protein kinase
MKK	MAPK kinase
MKKK	MKK kinase
MKP	Mitogen-activated protein kinase phosphatase
MYB	Myeloblastosis
NAC	NAM, ATAF and CUC
NAM	No apical meristem
NHX	Na^+^/H^+^ antiporter
NIN	Nodule inception
OSCA	Ca^2+^ permeable stress-gated cation channels
PA	Phosphatidic acid
PIP QTL	Plasma membrane intrinsic protein Quantitative trait locus
RAV	Related to ABI3 and VP1
RLK	Receptor-like kinase
ROS	Reactive oxygen species
SIT	Salt intolerance
SOD	Superoxide dismutase
SOS	Salt overly sensitive
TCP	Teosinte branched 1/cycloidea/proliferating cell
TF	Transcription factor
TIP	Tonoplast intrinsic
TPS	Trehalose-6-phosphate phosphatase
TPP	Trehalose-6-phosphate synthase
TRX	Thioredoxin
VP	Vacuolar-type H^+^-pyrophosphatase
ZF	Zinc finger
